# Impact of Sugary Food Consumption on Pregnancy: A Review

**DOI:** 10.3390/nu12113574

**Published:** 2020-11-22

**Authors:** Rosa Casas, Sara Castro Barquero, Ramon Estruch

**Affiliations:** 1 Department of Internal Medicine, Hospital Clinic, Institut d’Investigació Biomèdica August Pi i Sunyer (IDIBAPS), University of Barcelona, Villarroel, 170, 08036 Barcelona, Spain; sacastro@clinic.cat (S.C.B.); restruch@clinic.cat (R.E.); 2 CIBER 06/03: Fisiopatología de la Obesidad y la Nutrición, Instituto de Salud Carlos III, 28029 Madrid, Spain

**Keywords:** pregnancy, gestational diabetes, simple sugar, preeclampsia, obesity, metabolic programming

## Abstract

Obesity in pregnancy has been directly associated with an increased risk of almost all pregnancy complications such as gestational hypertension, preeclampsia, gestational diabetes mellitus (GDM), and premature delivery. Thereby, according to current evidence available, life-style interventions to prevent pre-pregnancy overweight and obesity in women of fertile age are necessary to reduce the negative impact of obesity on mother and child health. Unhealthy dietary patterns, together with the increased consumption of processed foods rich in simple sugar and sweeteners are some of the responsible, among others, for the increase in obesity rates during the last years. Nevertheless, how its consumption can affect pregnancy outcomes and long-term children’s health is still uncertain. This review aims to collate the available evidence about the consequences of unhealthy dietary patterns and sugary products consumption, including sweeteners, during pregnancy for obesity in childhood and mid-childhood. High simple sugar intake during gestation may contribute to an excessive gestational weight gain (GWG) as well as to develop other pregnancy complications such as GDM, preeclampsia and preterm birth. The heterogeneity of study populations, sample size, different approaches to measure GWG, GMD, preeclampsia, and birth weight, among other conditions, might explain the divergences observed among studies. Therefore, large, well-designed intervention-controlled trials with biological biomarkers to ensure dietary adherence are necessary to evaluate the effectiveness of lifestyle interventions in order to provide effective nutritional advice.

## 1. Introduction

It is well documented that the prevalence of obesity among children and adolescents has doubled around the world in the last 30 years [1]. Currently, one out of three children is overweight or obese [2], leading to possible cardiometabolic disturbances, mental health disorders and obesity during adulthood [3,4]. In addition, obesity has also been linked to some types of cancer and even arthritis [5]. Therefore, obesity comprises a large number of multifactorial problems, including high blood pressure, insulin resistance and type 2 diabetes mellitus (T2DM), high cholesterol concentrations, fatty liver disease, asthma, sleep apnea and joint pain, among others [6].

In most cases, obesity is the result of an excessive intake of calories that the body stores as fat. This excess of energy mainly comes from foods rich in fat and sugar [6]. According to the World Health Organization, free sugars, including added sugars, should be limited to less than 10% of daily calories [7]. The Dietary Guidelines for Americans (2015–2020) also recommends that added sugar should be reduced to less than 10% of total energy [8]. However, the results of 11 European surveys published by Azaïs-Braesco et al. [9] showed that relative intakes of sugar were higher in children (from 16 to 26% of total energy intake for both sexes) than in adults (from 15 and 21% of total energy intake for both sexes). Moreover, the National Health and Nutrition Examination Survey (NHANES) of 2003–2004 to 2011–2012 [10], showed that consumption of added sugars in absolute grams among non-pregnant women was 76.7 g, being higher for pregnant women at 85.1 g. Sugar-sweetened beverages (SSB), cakes, cookies, and pastries, sugars and sweets, juice drinks and smoothies, and milk desserts were the main sugary foods chosen by pregnant and non-pregnant women.

**Figure 1:**
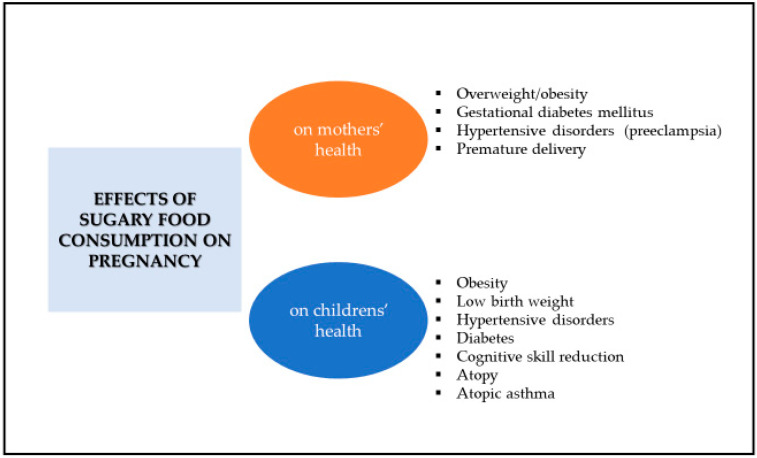
Effects of sugary food consumption in pregnancy on mothers’ and childrens’ health.

First, several observational studies and meta-analyses, both in adults and children, have reported that consumption of sugary foods, especially SSB, is related to weight gain, obesity, metabolic syndrome, and T2DM [11,12,13]. Several clinical studies have reported that higher consumption of added sugars from SSB is associated with unhealthy lifestyles, poor-quality dietary patterns and greater total energy intake, which might explain weight gain, gestational diabetes mellitus (GDM), hypertensive disorders and premature delivery, among other conditions (Figure 1) [14,15,16,17,18,19,20]. Second, there is a large amount of clinical evidence showing that following a healthy dietary pattern, which is by definition low in sugar-rich foods, such as the Mediterranean diet, can exert a beneficial influence on adverse gestational and birth outcomes [21,22]. Indeed, several studies have confirmed that a healthy dietary pattern during pregnancy reduces the incidence of hypertensive disorders [21], GDM [23], premature birth [22,24] and low birth weight [25].

Third, it has been well demonstrated that being obese before pregnancy and during gestation increases the risk of obesity in childhood, adolescence, and adulthood. Thus, some authors have pointed out that the consumption of sugary foods during gestation might exert a certain influence on intrauterine programming [26,27]. Thereby, it seems clear that obesity has its origin in early life.

The purpose of this review was to investigate the possible relationship between sugary food consumption during gestation and obesity in childhood and mid-childhood, as there is currently scarce documented information, with a simple bibliographic review of the literature published in the last 20 years, evaluating humans, adults (>18 years) and written in Spanish or English. The bibliographic search was performed through PubMed, ScienceDirect, and Google Scholar from June 2020 to September 2020. The keywords used for this search were: pregnancy, gestational diabetes mellitus, premature birth and low birth weight, preeclampsia, obesity and metabolic programming. We also investigated whether the impact of high sugary food intake on maternal health (excessive weight gain) is associated with complications during pregnancy and the impact on the fetus.

## 2. Cravings during Pregnancy

During pregnancy, women report food cravings and aversions, which may lead to choosing to eat certain unhealthy foods [28,29,30,31]. It is estimated that between 50–90% of pregnant women have one or more food cravings during gestation [32]. These unhealthy foods often provide excess energy intake, which leads to gestational weight gain (GWG) and the development of obesity in pregnancy [32]. On the contrary, aversions are associated with limiting or avoiding the intake of certain foods because of their association with vomiting and nausea [28]. To date, the mechanisms underlying food cravings are unknown, although physical and hormonal changes during pregnancy might play a key role [31]. Additionally, some authors have suggested that energy requirements during pregnancy are increased, which might lead to having a preference for candies and sweet foods [29]. In fact, Belzer et al. [33] reported that US women with mild GDM without dietary restrictions (e.g., weight-loss oriented dietary advice or low-sodium diets) showed a higher preference for this type of food. The most commonly craved foods during pregnancy seem to be dairy products (ice cream) and sweet foods (chocolate, fruit and fruit juice) and, in a lower proportion, salty foods (chips) [34].

A prenatal healthy dietary pattern is essential to avoid adverse gestational and birth outcomes [34]. Therefore, cravings for sugary foods are far from being considered part of a healthy diet.

## 3. Sugar Consumption and Pregnancy Complications

**Figure 2:**
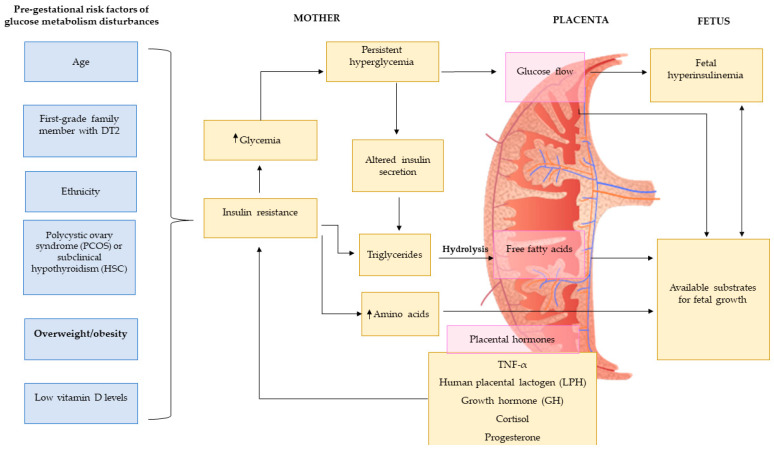
Pre-gestational risk factors of glucose metabolism disturbances. Figure adapted from Agha-Jaffar et al. [26].

There is a large amount of evidence showing that sugar intake during pregnancy is directly associated with GWG and the development of several pregnancy complications such as GDM, preeclampsia and preterm birth (Figure 2). Below, we discuss how added sugars, sugary products, intrinsic sugar and SSB intake can impact maternal health during pregnancy.

A total of 39 studies were selected for the final evaluation; some of the studies evaluated two or more outcomes, thus overlapping in the results. The study characteristics are summarized in Table 1.

### 3.1. Weight Gain during Pregnancy

On one hand, it is clear that during pregnancy there is a steady stream of glucose from the mother’s placenta to the fetus, being the main energy substrate for intrauterine growth [65]. On the other hand, adequate GWG is also important to ensure the healthy growth and development of the fetus [66]. According to the Institute of Medicine, obese women should limit their GWG to 5–9 Kg [67]. Nevertheless, among 1,309,136 pregnant women of US analyzed, it was estimated that close to 50% of pregnant women showed a higher than recommended GWG [66]. Currently, the prevalence of weight gain and obesity is increasing in the obstetric population by more than 40 and 50%, respectively [68,69].

It is necessary to highlight that excessive gestational weight is associated with adverse effects during gestation, such as small or large fetus for gestational age, macrosomia, cesarean delivery, preeclampsia, gestational hypertension, preterm birth, small or large size for gestational age at birth or offspring obesity [66,70]. Nevertheless, up to now, few studies have investigated the possible direct association between sugary food intake and GWG. 

According to the last Cochrane systematic review including 27 studies and 3964 women [35], there is no evidence that a behavioral intervention based on the promotion of healthy dietary habits and regular physical activity can prevent GWG during gestation. However, the results obtained in several studies have reported that exercise and caloric restriction in obese pregnant women might prevent GWG. A meta-analysis that included 36 randomized trials with 12,526 women [36] showed significant reductions of GWG in obese women that followed-up behavioral interventions (diet and physical activity).

A few interventional studies have also described this association [18,37,38,39]. In a randomized controlled trial (RCT) carried out by Poston et al. [37] in 1555 obese pregnant women (mean body mass index, BMI = 36.3 kg/m^2^, 15–18 weeks plus 6 days of gestation, and ≥16 years old), the women were randomized into two study arms: a behavioral intervention and standard antenatal care (control group). After 8 health trainer-led sessions, the authors reported that the women in the intervention group had less GWG over the total pregnancy than the control group, although the primary outcomes did not differ between groups (25% vs. 26%, respectively; *p* = 0.68). In another RCT [38], 61 pregnant women (BMI > 25) in the first trimester were included and randomized into two study arms: a Therapeutic Lifestyle Changes (TLC) Program and a control group. The TLC Program included changes in diet (overweight: 1700  kcal/day, obese: 1800  kcal/day) and mild physical activity (30 min/day, 3 times/week). After the intervention, the authors reported that women assigned to the TLC group showed less GWG (6.7  ±  4.3  kg) compared to controls (10.1  ±  5.6  kg, *p*  =  0.047). Similar results were reported by Renault et al. [39], who showed that only the consumption of added sugar was associated with GWG. Furthermore, the sugary foods most strongly associated with weight gain were sweets, snacks, cakes, and soft drinks. Women who consumed ≥ 2 units/day of sweets showed a 5.4 kg greater weight gain than those with a low (<1 week) sweet intake. Finally, Wattar et al. [18] carried out an RCT including 1252 pregnant women with metabolic risk factors. Of these, 593 women were randomly allocated to Mediterranean-style diet intervention. The authors reported a lower GWG in the intervention group (mean 6.8 versus 8.3 kg; adjusted difference −1.2 kg, 95% confidence interval [CI] −2.2 to −0.2, *p* = 0.03) compared to the control group. 

Several observational studies have also reported associations between sugar intake during gestation and excess maternal weight gain [40,41,71,72,73]. A large, prospective, Danish, cohort study, which included 46,262 pregnant women, found a strong association between added sugar intake and GWG (Q5 vs. Q1: 34, 95% CI 28 to 40 g/week, *p* for trend <0.0001), with an average extra weight gain of 1.4 kg during pregnancy. On the other hand, a higher protein/carbohydrate ratio was related to lower GWG, possibly because of decreased added sugar intake [42]. Additionally, Diemert et al. [40] found that 60% of pregnant women (N = 200) gained more weight than recommended. Saturated fat and sugar were among the nutrients that most contributed to total energy consumption. There was a positive correlation (*p*  =  0.006) between weight gain and monosaccharides and saccharose. Similar results were reported by Olafsdottir et al. [41] after analyzing 495 pregnant women in Iceland. The authors reported that women who ate more sweets in early pregnancy increased the risk of gaining excessive weight (OR = 2.52, CI =1.10–5.77, *p* = 0.029). After analyzing 3360 pregnant Finnish women, Uusatilo et al. [43] also reported, that adherence to a higher fast-food dietary pattern, characterized by high consumption of hamburgers, pizza, sweets, soft drinks and added sugars, was strongly associated with GWG. Despite the fact that it is an observational study, causation cannot be proved, while the results supported that frequent consumption of fast foods and snacks might influence excessive GWG. Thus, recent evidence has shown that unhealthy dietary patterns are correlated with excessive GWG [43,71,72,73].

### 3.2. Sugar Consumption and Gestational Diabetes

Up to 16% of pregnant women are diagnosed with GDM during pregnancy [74]. For women with GDM, progression to T2DM is estimated to be between 15 to 50% at 5 years [75]. At present, the prevalence of GDM is rising because of the high incidence of both overweight and obesity around the world [76], highlighting that weight gain is a significant predictor of T2DM at 15 years of follow-up [77]. In the short- and long-term, GDM is associated with serious obstetric and neonatal complications during gestation and childbirth, including an increased risk for both the mother and child. Some examples are macrosomia, birth injury, cesarean delivery, offspring obesity, epigenetic changes in children with a higher predisposition to both obesity and T2DM in adulthood, etc. [66,70,78]. 

There is robust evidence that reinforces how a healthy dietary pattern such as the Mediterranean diet can reduce the incidence of GDM during pregnancy [36,79,80,81,82,83]. One of the main variables directly related to the intake of sugary foods is GWG which might also be a predictor for GDM [17,84,85]. Scientific evidence supports that a diet rich in simple sugars might decrease insulin sensitivity and insulin secretion [86,87]. 

In this sense, Mijatovic-Vukas et al. [17] carried out a systematic review including the data of 30,871 pregnant women. The authors reported significant associations between SSB and the risk of GDM (RR for pregnant women ≥5 weeks = 1.23, 95% CI: 1.05–1.45, *p*-value = 0.005). After considering different sub-types of SSB, the highest association with GMD was observed for sugar-sweetened cola (RR high vs. low intake = 1.29, 95% CI: 1.07–1.55). No significant associations were observed for non-cola SSB (RR high vs. low = 0.99, 95% CI: 0.78–1.25). Wattar et al. [18] reported a reduction in the odds of GDM by 35%. The same authors designed a meta-analysis of RCTs, which included 2 trials and 2397 pregnant women who followed a Mediterranean diet supplemented with nuts and extra virgin olive oil, where authors reported a significant reduction in GDM (OR = 0.67, 95% CI 0.53–0.84, I2 = 0%). 

Nevertheless, RCTs based on behavioral interventions (changes in eating behavior and promotion of physical activity) have shown contradictory results in GDM prevention [18,37,38,39]. On one hand, some authors showed that energy restriction plus the promotion of regular physical activity was associated with improved pregnancy complications, such as GDM, gestational hypertension and preterm delivery in obese women [38]. In contrast, Poston et al. [37] observed that the incidence of GMD in obese women was similar between the participants assigned to the intervention group and the control group (26 and 25%, respectively). In addition, Wattar et al. [18] reported a lower risk of GDM (35%) in pregnant women who present metabolic risk factors (obesity, chronic hypertension, or hypertriglyceridemia) but followed a Mediterranean-style diet.

Finally, the results obtained after analyzing 253 pregnant women (aged between 16 to 41 years) from the NHANES survey showed that women who followed a diet rich in added sugar and viscera; low fruits, vegetables and seafood had a higher risk of developing GDM than those with a diet based on a high intake of nuts, seeds, fat and soybean and low milk and cheese intake [44]. Additionally, in a prospective study that included 13,475 US women pre-pregnancy, 860 incident GDM cases were identified after 10 years of follow-up. Furthermore, the authors reported that pre-pregnancy women with a sugar-sweetened cola consumption ≥ 5 servings per week had a higher risk of GDM (22%) compared to those with a consumption of less than 1 serving/month [15]. In this study, the authors did not include juice intake in the analysis. Another study, the “Seguimiento Universidad de Navarra” (SUN) cohort, also evaluated SSB consumption and the risk of developing GDM [45]. In this case, the authors followed 3396 pre-pregnancy women over 10 years. During this period, 172 new cases of GDM were diagnosed, and the authors reported that SSB consumption was strongly associated with a higher risk of GDM when they became pregnant (OR = 2.03, 95% CI: 1.25–3.31). Nevertheless, they found no association between sugar-free soft drink intake and the risk of GDM. A prospective Canadian study [46] including 205 women with singleton pregnancies without type 1 or type 2 diabetes found that adding sugar to coffee and tea was directly associated with a higher risk of hyperglycemia.

### 3.3. Sugar Consumption and Preeclampsia

Preeclampsia can be defined as a disorder during pregnancy characterized by hypertension and often proteinuria in healthy women [88]. It is a common pregnancy complication and affects between 2 to 8% of pregnancies worldwide [89]. In addition, it is one of the most common causes of morbidity and mortality in both pregnant women and their offspring [88,90]. Both type 1 and 2 diabetes can further increase the risk of preeclampsia [91]. 

Although the known risk is associated with preeclampsia, the number of studies that correlate the consumption of sugary foods with preeclampsia risk during pregnancy is limited [47,48,49,82,92].

In a prospective Norwegian study that included 32,933 normal and overweight pregnant women, a high intake of SSB (≥125 mL/day) was associated with a higher risk of preeclampsia (OR = 1.27, 95% CI: 1.05, 1.54) [91], while a high intake of intrinsic sugars (such as dried and fresh fruit) was associated with a lower risk (OR = 0.79, 95% CI: 0.67, 0.93 and OR = 0.79, 95% CI: 0.68, 0.92, respectively). The authors also reported that high intake of sugar-sweetened beverages in women with a BMI < 25 showed a stronger association with the risk of preeclampsia than those with a BMI ≥ 25 (OR 1.32 v. 1.28, respectively). Moreover, Clausen et al. [49] reported that the risk of preeclampsia was directly associated with high sucrose intake (>25% of total energy) after analyzing 3133 pregnant Norwegian women. The NHANES survey showed that every 12 oz. (~354 mL) of SBBs was associated with a reduction of 2.3 points of the Alternate Healthy Eating Index modified for Pregnancy (AHEI-P) score, which measured the quality of the diet. SSB intake was also associated with an intake of 124 more calories [16]. The authors estimated that SSB consumption, which was set at 0, should be an average AHEI-P of 6.4 and with an average total calorie intake less than 203.5. Brantsaeter et al. [21] investigated the association between different dietary patterns during pregnancy and the risk of preeclampsia in 23,423 pregnant Norwegian women. They showed that an unhealthy pattern, characterized by high consumption of processed meat, salty snacks, and sweet drinks, was strongly associated with an increased risk of preeclampsia [OR for tertile 3 vs. tertile 1: 1.21; 95% CI: 1.03, 1.42]. However, the authors considered their results could be influenced by non-included confounding factors in the analysis, thereby a causal inference between dietary habits and the risk of preeclampsia should be evaluated. A prospective longitudinal cohort study [19], including 55,139 Danish women, found a harmful association between following a Western diet and pregnancy-associated hypertension (PAH) which included gestational hypertension (GH) and preeclampsia. Concretely, a higher adherence to a Western diet was associated with a higher risk for GH (OR = 1.18; 95% CI 1.05–1.33) and preeclampsia (OR = 1.40; 95% CI 1.11–1.76). The authors did not find any significant associations between a diet high in sugary products and GH (OR = 1.05; 95% CI 0.94–1.16) or preeclampsia (OR = 1.10; 95% CI 0.90–1.35). Similar results were reported by Schoenaker et al. [48], who analyzed 3582 women participating in the 9-year Australian Longitudinal Study on Women’s Health and found that a Mediterranean-style dietary pattern was inversely related to the risk of developing PAH compared to three other dietary patterns (1. based on meat, high-fat, and sugary foods; 2. based on fruit and low-fat dairy; and 3. based on cooked vegetables). However, the authors found no significant association between SSB and PAH.

## 4. Sugar Consumption and Birth Outcomes

Recent scientific evidence postulates that the mother’s weight gain (pre-and during pregnancy) increases the risk of obesity in the child in early and middle childhood, as well as in adulthood [93,94].

It is a fact that both glucose and fructose can diffuse throughout the maternal placenta, which can affect fetus development [95]. The mechanisms underlying these harmful effects are, on one hand, that the consumption of fructose *per se* might be linked to obesity [95], and on the other hand, it is well established that the likelihood of developing childhood metabolic disorders is higher in children of obese mothers or that have developed GDM during pregnancy. Thus, it seems relevant to investigate how sugary food intake contributes to modifying the metabolic profiles of offspring during pregnancy.

### 4.1. Sugar Consumption and Premature Delivery

Preterm delivery (before 37 weeks of gestation) is one of the main causes of morbidity and almost 75% of neonatal mortality in the short and long-term [22,96]. According to a meta-analysis [97], the rate of preterm delivery in European countries was around 5% and increase up to 18% in some African countries in 2010.

The relationship between SSB intake and premature delivery has been studied. Although there is limited evidence, some results obtained up to now suggest a strong association between SSBs consumption and increased risk of premature delivery.

Several studies have shown associations between maternal diet and preterm delivery [22,24,82,98,99]. In this sense, Englund-Ögge et al. [22] analyzed the maternal dietary pattern of 66,000 pregnant Norwegian women and its association with premature delivery. A balanced diet, characterized by high consumption of vegetables, fruits, oils, water, whole grain cereals and fiber-rich bread was associated with a lower risk of preterm delivery compared to women who adhered to a Western diet. Similar results were observed for the New Nordic Diet (NND) after studying 59,949 pregnant Norwegian women [24]. In this case, the NND showed strong protection against preterm birth (OR = 0.77; 95% CI 0.66–0.89).

Recently, soft drinks (both artificially and sugar-sweetened) have been linked to an increased risk of premature delivery [20]. In this study, 59,334 Danish women were analyzed, who reported dietary information about their soft drink daily intake at around 25 weeks of pregnancy. In this case, a significant association was observed between soft drink intake and the risk of preterm delivery (*p* for trend <0.001; for both soft drinks). Moreover, women who drank one serving of artificially sweetened carbonated soft drinks per week showed a 38% greater risk of preterm delivery compared to women who did not drink artificially sweetened soft drinks. On the other hand, women with a consumption ≥ 4 servings of artificially sweetened carbonated soft drinks per week showed an increased risk of 78%, which was observed in both normal-weight and overweight women [20]. Similar results were observed by Englund-Ogge et al. [50], who reported that a high intake of soft drinks (artificially and sugar-sweetened beverages) was positively associated with a higher risk of preterm delivery. Among the 60,761 pregnant women studied, preterm delivery occurred in 3281 (5.4%) cases. In fact, the authors estimated that drinking more than 1 serving per day of artificially sweetened beverages increased the risk by 11%, whereas the risk increased up to 25% when the consumption was of sugar-sweetened beverages. In addition, in an English cohort of 8914 pregnant women, Petherick et al. [51] found that women who drank more than 4 servings of SSBs (cola) per day showed a higher risk of preterm delivery. However, contrary to the studies cited before, the authors did not find associations between the daily consumption of artificially sweetened beverages and the risk of preterm delivery.

### 4.2. Offspring Weight

A meta-analysis that included 162,129 mothers and their children from 37 weeks of pregnancy to delivery from several cohort studies in Europe, North America, and Australia, investigated the effect of excessive GWG on the development of offspring obesity [52]. The authors reported that both maternal overweight and obesity pre-pregnancy and excess weight gain during pregnancy are associated with a higher risk of presenting overweight/obesity during childhood. It was estimated that 21.7% of overweight and 41.7% of obese children could be attributed to maternal weight, while 11.4% of overweight and 19.2% of obese children can be attributed to GWG [52]. In addition, a systematic review [53] concluded that following a healthy diet, such as a Mediterranean diet, during pregnancy, together with a reduction of refined carbohydrate intake, might have a positive effect on offspring adiposity between 6 and 18 months after birth. Nevertheless, the authors also reported inconclusive or null findings associations of n-3 polyunsaturated fatty acids, protein, SSB artificially sweetened beverage intake and offspring body size.

However, up to now, few interventional studies have investigated whether lifestyle changes focused on the promotion of a healthy diet and physical activity during pregnancy, showed protective effects in large-for-gestational-age infants (≥90th birthweight centile) [37]. In this sense, Poston et al. [37] did not observe differences between groups after a behavioral intervention in the UK Pregnancies Better Eating and Activity Trial. They observed a similar number of infants with increased weight for gestational age in both groups (8% in the standard care group vs. 9% in the intervention group). Neither were differences in adverse events (including neonatal death), fetal death in utero and maternal deaths found, or in the number of small-for-gestational-age infants (≤5th birthweight centile) or the incidence of miscarriage. Similar results were reported by the Australian LIMIT study [54]. In this case, 1108 overweight/obese pregnant women were randomized into an intervention group, the aim of which was to change unhealthy dietary habits and increase physical activity levels while the remaining 1104 pregnant women included were randomized into a control group (standard care). The authors reported that the risk of delivering large-for-gestational-age babies in women assigned to the intervention arm was not reduced in comparison to the control arm (RR = 0.90, 95% CI: 0.77 to 1.07; *p* = 0.24).

Several observational and prospective studies have also shed light on this association. Phelan et al. [55] observed that following an unhealthy dietary pattern, mainly based on sweets and processed food intake, in overweight and obese mothers (n = 132) has important effects on child weight. A high sugary food intake in overweight and obese mothers in early pregnancy was the strongest predictor of large-for-gestational-age infants (β = 0.19; *p* = 0.004), macrosomia (OR = 1.1; 95% CI: 1.0–1.2; *p* = 0.004), high birth weight (>90th percentile at birth) (OR = 1.2; 95% CI: 1.1–1.3; *p* = 0.002) and weight at 6 months (β = 0.30; *p* = 0.002) for each 1% increase in energy consumed from sweets. Moreover, for mothers with a normal weight (n = 153) during pregnancy, higher consumption of soft drinks was the strongest predictor of weight at birth (β = 0.16; *p* = 0.04) but not at 6 months. A study carried out in Singapore [56] that included 910 offspring, reported that higher consumption of sugar and refined carbohydrates during late pregnancy was associated with a higher infancy BMI z-score (2–4 years). Quah et al. [57] reported that increments of 100 mL of SSB per day were linked to a higher BMI, a sum of skinfolds and risk of overweight/obesity in middle childhood (at age 5–6 years). The Project Viva [58], a prospective pre-birth cohort study, which includes 1078 mothers and their infants, reported that higher consumption of SSB (0.6 servings/day) in the second trimester of pregnancy was associated with a higher risk of adiposity in middle childhood (median age of 7.7 years). Thus, for each additional serving per day of SSB, the authors reported a higher BMI z score (0.07 U; 95% CI: −0.01 to 0.15), fat mass index (0.15 kg/m^2^; 95% CI: −0.01 to 0.30), sum of subscapular and triceps skinfold thickness (0.85 mm; 95% CI: 0.06 to 1.64), and waist circumference (0.65 cm; 95% CI: 0.01 to 1.28). In a study of 3312 mothers and their offspring, Jen et al. [59] also reported that SSB intake during gestation was strongly associated with a higher BMI in 6-year-old children and especially with a higher percentage of fat mass.

Furthermore, similar results have been reported in the case of artificially sweetened beverage intake. A recent publication [60] including 1257 pregnant mothers and their infants showed that maternal dietary pattern based on fried foods and SSB during pregnancy was associated with a higher risk of having an infant in the rising-high BMI trajectory group (OR = 1.32; 95% CI: 1.07–1.62; *p* = 0.008), as well as becoming overweight/obese children at 4 years of age (OR = 1.31; 95% CI: 1.11–1.54; *p* = 0.001).

The Canadian Healthy Infant Longitudinal Development (CHILD) Study carried out in 3033 mothers (32.7 ± 4.7 years and 24.8 ± 5.4 kg/m^2^) and their infants with an 89% follow-up showed that the children of mothers who consumed artificially sweetened beverages daily had a 2-fold higher risk of developing overweight in early childhood (1 year) than those with mothers who did not consume these beverages [61]. In addition, the daily consumption of artificially sweetened beverages was associated with a 0.20-unit increase in the BMI z-score in infants. The Danish National Birth Cohort including 918 mothers and their infants followed at birth and at 5 and 12 months and at 7 years studied the effects of SSB and artificially sweetened beverage consumption during pregnancy and the risk of offspring obesity [62]. The children of women reporting daily consumption of artificially sweetened beverages showed a higher risk of being large-for-gestational-age (RR = 1.57; 95% CI: 1.05, 2.35 at birth) and becoming overweight or obese after 7 years (RR = 1.93; 95% CI; 1.24, 3.01). Interestingly, the substitution of artificially sweetened beverages by water showed a lower RR of overweight and obesity at 7 years (RR = 0.83; 95% CI: 0.76, 0.91). However, when artificially sweetened beverages were substituted by SSB, a higher risk of overweight and obesity at 7 years was observed (RR 1.14; 95% CI: 1.00, 1.31). 

### 4.3. Other Disorders

It is known that increased plasma levels of ceramides are related to cardiovascular disease and the pathogenesis of T2DM. In a cross-sectional study [100] it was observed that cumulative SSB intake (1 serving/day) might contribute to an increased risk of cardiometabolic diseases, with a positive correlation being found between SSB intake and three circulating ceramide concentrations.

According to a cohort study published in the American Journal of Preventive Medicine including 1234 mothers–child pairs recruited in pregnancy and early childhood, there is a strong association between SSB (including artificially sweetened carbonated soft drinks) and child health (median ages 3.3 and 7.7 years). In addition, the authors reported that an excess of sugar intake (mean 49.8 g/day) during pregnancy might lead to reduced cognitive skills in children such as mid-childhood Kaufman Brief Intelligence Test (KBIT-II) non-verbal scores and early or mid-childhood cognition scores [63]. On the contrary, high consumption of fruit was associated with improved cognition in early childhood. These improvements might be linked to the phytochemical content in fruit and not with fructose itself, since fruit juice intake was not associated with improved cognition.

Finally, in a study of 8956 pregnant women, Bédard et al. [64] analyzed the association between free sugar intake during pregnancy and asthma, wheezing, hay fever, eczema, atopy, total serum IgE and lung function in mid-childhood (7–9 years). A higher intake of free sugar during pregnancy was linked to a higher risk of atopy (OR = 1.38, 95%, CI: 1.06–1.78, per quintile *p*-trend = 0.006) and atopic asthma (OR = 2.01, 95% CI 1.23–3.29; per quintile *p*-trend = 0.004) in mid-childhood.

## 5. Limitations

Firstly, to differentiate specific types of sugars in order to evaluate their health effect is complex. Sugary foods include beverages, concretely SSBs, sugar added or sweetened products or derivatives, which intake is high in some dietary patterns, such as Western diet. Therefore, the health effects observed might be the consequence of reverse causality. Furthermore, residual confounding cannot be excluded from observational studies. Few RCTs have been performed to date. Most of the prospective cohort studies were based on Food Frequency Questionnaires (FFQ) or 24-h dietary recall, which were self-reported and might lead to measurements error. Moreover, under- and over-reporting may have occurred in FFQ. It is necessary to highlight that unhealthy foods are usually underreported in comparison to those foods consider as healthy, which might explain why some studies did not find a significant association between dietary outcomes (such as added sugars) and diseases (such as GDM or preeclampsia). Estimating sugar intake is complex due to it is expressed as added, free and/or total sugars and these values may differ among countries. Moreover, we reported diverse effects among studies for sweetened beverage intake. These different health effects observed can be associated with the type of sweetener (natural or artificial), dosages and intensities used. Additionally, its quantification is very difficult because the amounts used in food production are not always reported.

On the other hand, some studies obtain self-reported pre-pregnancy weight, limiting the validity of the GWG assessment. In other studies, gestational week for the reported GWG was not defined while others used excessive GWG early in the second trimester instead of late second trimester GWG to predict GDM. In addition, diet during pregnancy could be modified among pregnancy trimesters. Another potential limitation is the population studied, healthy nulliparous low-risk women, while others include pregnant women with metabolic syndrome criteria, overweight and/or obesity or with diabetes. 

## 6. Conclusions

Diet is a modifiable risk factor that plays a key role in pregnancy and future child health. Unbalanced dietary patterns rich in simple sugars and processed foods are clearly associated with the main complications during pregnancy, such as GDM, PAH and excess GWG. Moreover, following an unhealthy dietary pattern during pregnancy not only has an impact on the mother with an increased risk of metabolic disturbances, but the health of her child can also be compromised, with an increased risk of becoming overweight or obese during middle childhood, increased weight for gestational age, higher adiposity, a worse metabolic profile and declined cognitive skills. 

The heterogeneity of the study populations (high-risk vs. low-risk pregnant women), limited sample sizes, different nutritional outcomes (high intake of simple sugars, SSBs consumption, sugary foods consumption, unbalanced diet among other dietary components), measurement of dietary adherence, the lack of physical activity records and the different approaches used to measure GWG, GMD, preeclampsia, and birth weight, among other conditions, could explain the divergences observed among studies. Therefore, well-designed intervention controlled trials, with large sample size and biological biomarkers to ensure dietary adherence are necessary to evaluate the effectiveness of the interventions carried out.

## Figures and Tables

**Table 1:** Summary of study characteristics, outcomes and main results of studies included in the analysis.

Reference	Design, Subjects (n)	Population	Intervention/Method	Primary Outcome	Main Results
Chen et al. [15]	Prospective study, n = 13,475	Women from the Nurses’ Health Study II, 24–44 years, who reported having at least one singleton pregnancy lasting 6 months or more.	Semiquantitative FFQ	Gestational diabetes mellitus	Intake of sugar-sweetened cola (≥5 servings/week) was positively associated with the risk of GDM (RR = 1.22; 95% CI 1.01–1.47). No significant association between SSB intake and “diet” beverages was found with the risk of GMD.
Gamba et al. [16]	Cross-sectional study from NHANES survey, n = 1154	Pregnant women that completed dietary data	AHEI-P to measure diet quality 24-h dietary recalls	Diet quality and total energy intake	Every 12 oz. of SSBs consumed was associated with a 2.3 lower AHEI-P score (95% CI 1.6–2.9) and the consumption of 124 more calories.
Mijatovic-Vukas et al. [17]	Meta-analysis, 40 studies, n = 30,871	Women with available diet or PA data during pre-pregnancy/early pregnancy	Different dietary patterns or PA	Gestational diabetes mellitus	Higher SSB intake was associated with higher risk of GDM (RR ≥ 5 week = 1.23; 95% CI 1.05–1.45, *p*-value = 0.005). The strongest association was observed for sugar sweetened cola (RR high vs. low intake = 1.29; 95% CI 1.07–1.55) but not for non-cola SSB (RR high vs. low = 0.99; 95% CI 0.78–1.25).
Wattar et al. [18]	Multicenter, RCT, n = 1252 Meta-analysis, 2 RCT, n = 2397 women	Women with metabolic risk factors, ≥16 years, <18 weeks’ gestation	Mediterranean-style diet and usual care diet for meta-analysis	Gestational diabetes or preeclampsia and offspring (stillbirth, small for gestational age, or admission to neonatal care unit)	The risk of gestational diabetes was reduced in Mediterranean-style diet group compared to usual care diet (adjusted OR = 0.65; 95% CI 0.47–0.91, *p* = 0.01), and gestational weight gain (mean 6.8 versus 8.3 kg). Meta-analysis showed a significant reduction in the risk of gestational diabetes (−33%).
Ikem et al. [19]	Prospective longitudinal study, n = 55,139	Pregnant Danish women, in the ~25-week gestation	Validated semi-quantitative FFQ	Gestational hypertension and preeclampsia.	Western diet (high in fast food, added sugar and saturated fats) increased the risk of GHD (OR 1.18; 95% CI 1.05–1.33) and PE (OR 1.40; 95% CI 1.11–1.76). No significant association between sugary products and GHD was observed (OR = 1.05; 95% CI 0.94–1.16) or PE (OR = 1.10; 95% CI 0.90–1.35)
Halldorsson et al. [20]	Prospective cohort study, n = 59,334	Pregnant Danish women, in the ~ 6–10-week gestation	Validated FFQ	Risk of preterm delivery	Significant association between soft drink intake and the risk of PTD (*p* for trend <0.001; for both soft drinks). Normal-weight and overweight women who drank one serving of ASB per week showed greater risk of preterm delivery (38%) compared to non-consumers. When the consumption was ≥4 servings of ASB per weeks the risk increased by 78%
Brantsaeter et al. [21]	Prospective cohort study, n = 23,423	Nulliparous pregnant women from the Norwegian Mother and Child Cohort Study (MoBa). Gestational age: 22 weeks	Semiquantitative FFQ	Preeclampsia	Processed meat, salty snacks, and sweet drinks were strongly associated with higher risk of preeclampsia (OR for tertile 3 vs. tertile 1 = 1.21; 95% CI 1.03–1.42).
Englund-Ögge et al. [22]	Large prospective cohort study, n = 66,000	Pregnant Norwegian women with singleton pregnancies, without previously PTD, pregnancy duration between 22 + 0 and 41 + 6 gestational weeks, no diabetes, first enrolment pregnancy.	Validated FFQ	Risk of preterm delivery	A “prudent pattern” and a “traditional pattern” were associated with significantly reduced risk of PTD (HR = 0.88; 95% CI 0.80–0.97 and HR = 0.91; 95% CI 0.83–0.99) compared to women who adhered to a Western diet.
Rasmussen et al. [24]	Prospective, longitudinal cohort study, n = 59,949	Pregnant Danish women at 12 and 30 weeks of gestation	FFQ	Spontaneous and induced preterm birth (gestational age < 259 days (<37 weeks)).	Association between Western diet and induced PTD (OR = 1.66; 95% CI 1.30–2.11) and Western diet and spontaneous PTD (OR = 1.18; 95% CI 0.99–1.39) were observed comparing the highest vs. the lowest quintile.
Muktabhant et al. [35]	Systematic review of 27 RCTs or quasi-RCTs, n = 3964	Pregnant women with or without overweight and obesity. Gestational age: ≤20 weeks or >20 weeks.	Nutrition intervention, exercise intervention, health education or counselling	Gestational weight gain	Results were not statistically significant and consistent. Significant reduction for women that received behavioral counselling compared standard care (RR = 0.72; 95% CI 0.54–0.95).
i-WIP Collaborative Group [36]	Systematic review and meta-analysis of 36 RCTs, n = 12,526	Pregnant women (≥20 years.) with or without overweight and obesity.	Nutrition intervention, physical activity and mixed interventions	Gestational weight gain	Obese women that followed-up behavioral interventions based on diet and physical activity advice during pregnancy reduced gestational weight gain and decreased the risk of cesarean. No strong evidence was found for the effect of life-style interventions on individual offspring outcomes.
Poston et al. [37]	Multicenter, RCT, n = 1555	Obese pregnant women, 15–18 weeks plus 6 days of gestation and age >16 years.	Behavioural intervention or standard antenatal care	Gestational diabetes and large-for-gestational-age infants (≥90th customized birthweight centile)	No differences between groups were observed for the primary outcomes.
Petrella et al. [38]	Prospective, RCT, n = 61	Pregnant women with BMI >25 at first trimester and age >18 years.	No intervention or a TLC Program including diet (overweight: 1700 kcal/day, obese: 1800 kcal/day) and mild physical activity (30 min/day, 3 times/week).	Gestational weight gain, GDM, gestational hypertension, PTD	Gestational weight gain in obese women randomized to TLC was lower than control group (6.7 Kg vs. 10.1 Kg, *p* = 0.047). Lower incidence of GDM was observed in women randomized in TLC Program compared to the control group (23.3% vs. 57.1%, *p* = 0.009)
Renault et al. [39]	3-arm RCT, n = 342	Pre-pregnancy BMI ≥30 kg/m^2^, gestational age <16 weeks’ gestation, age >18 years	D + PA, PA and control	Gestational weight gain	Added sugar from foods appeared to be related to gestational weight gain (*p* for trend = 0.02). Sweets, snacks, cakes, and soft drinks were strongly associated with weight gain
Diemert et al. [40]	Prospective cohort study, n = 200	Healthy low-risk women (>18 years.), gestational age 12 + 0 to 14 + 6 weeks	Self-reported dietary intake	Gestational weight gain	Especially, overweight and obese women gained more weight than recommended. Saturated fat and sugar were the nutrients that most contributed to total energy consumption.
Olafsdottir et al. [41]	Observational study, n = 495	Pregnant women between 11 and 15 weeks	Semi-quantitative FFQ	Gestational weight gain	Higher intake of sweets during early pregnancy increased the risk of gaining excessive weight (OR = 2.52, CI 1.10–5.77, *p* = 0.029).
Maslova et al. [42]	Prospective cohort study, n = 46,262	Pregnant women with 6–10 weeks of gestation.	Complete data on dietary intake and GWG	Gestational weight gain	Added sugar consumption was strongly associated with GWG (Q5 vs. Q1: 34, 95% CI 28 to 40 g/week, *p* for trend <0.0001).
Uusatilo et al. [43]	Observational study, n = 3360	Fin women whose baby presented human leucocyte antigen-conferred susceptibility to type 1 diabetes. Recruited in 10th gestational week on average.	Validated FFQ	Gestational weight gain	“Fast food” dietary pattern (high in sweets, soft drinks, hamburgers, pizza and other fast foods) was positively associated with weight gain rate (kg/week)
Shin et al. [44]	Cross-sectional study, n = 253	Pregnant US women, from 16 to 41 years, included in the NHANES survey 2003–2012.	24 h dietary recall	Gestational diabetes mellitus	Pregnant women in the highest tertile of “high added sugar and organ meats; low fruits, vegetables and seafood” intake showed higher risk of GDM (OR 21.1; 95% CI 4.0–109.8) compared to those in the lowest tertile.
Donazar-Ezcurra et al. [45]	Prospective and dynamic cohort, n = 3396	Women that have notified at least one pregnancy between December 1999 and March 2012.	A validated 136-item semi-FFQ	Gestational diabetes mellitus	Consumption of ≥ 2 SSB servings/week was strongly associated with the risk of GDM at the beginning of pregnancy (adjusted OR: 2.03; 95% CI 1.25–3.31; *p* for trend: 0.006). There were no statistical associations between sugar-free soft drink intake and GDM risk.
Ley et al. [46]	Prospective Canadian cohort study, n = 205	Women with singleton pregnancies and without preexisting type 1 or type 2 diabetes. Aged ≥20 years and 24–28 week of gestation.	Validated FFQ	Gestational diabetes mellitus	Added sugar in coffee and tea were individually associated with increased fasting glucose (both *p* ≤ 0.02)
Borgen et al. [47]	Prospective Norwegian study, n = 32,933	Nulliparous women, in gestational weeks 18–22	A semi-quantitative FFQ	Preeclampsia	Sugar-sweetened carbonated and non-carbonated beverages (>= 125 mL/day) were significantly associated with higher risk of preeclampsia (OR = 1.27; 95% CI 1.05–1.54), both independently and combined compared to non-consumers.
Schoenaker et al. [48]	Australian Longitudinal Study on Women’s Health, n = 3582	Women were not pregnant at baseline (age: 25–30 years). Women who reported at least one live birth from different date of survey: 28–33 years, age: 31–36 y and age: 34–39 years Follow-up: 9 years	Validated FFQ	Hypertensive disorders of pregnancy	The Mediterranean-style dietary pattern was inversely associated with risk of developing hypertensive disorders of pregnancy (quartile 4 compared with quartile 1: RR = 0.58; 95% CI 0.42–0.81). No association was found between sugar dietary pattern and the risk of hypertensive disorders of pregnancy.
Clausen et al. [49]	Prospective, population-based cohort study, n = 3133	Norwegian pregnant women in the second trimester	Quantitative FFQ	Preeclampsia	Sucrose intake (>25% of total energy) was directly associated with the risk of preeclampsia (OR = 3.8, 95% CI 1.5–9.8, *p* = 0.01) compared with lower intake (≤8.5%).
Englund-Ögge et al. [50]	Large prospective cohort study, n = 60,761	Norwegian pregnant women at gestational weeks 17–18	Semiquantitative FFQ	Risk of preterm delivery	A high consumption of ASB and SSB (>1 serving/day) were associated with higher risk of preterm delivery (OR = 1.11; 95% CI 1.00–1.24 and OR = 1.25; 95% CI 1.08–1.45, respectively).
Petherick et al. [51]	Longitudinal multi-ethnic birth cohort study, n = 8914	Pregnant women at 26–28 weeks of gestation at which time a baseline questionnaire was completed.	Consumption of ASB (cola) and SSB (cola): none, one, two, three or four or >4 cups per day (each cup measuring 200 mL).	Risk of preterm delivery	No relationship was observed between daily AS cola beverage consumption and PTD. Women who drank ≥4 cups per day of SS cola beverages had higher risk of PTD compared to non-consumers or <1 cup per day participants.
Voerman et al. [52]	Meta-analysis of 37 cohorts, n = 162,129 mothers and their children	Mothers with singleton live-born, before 20 weeks of gestation, that had information available on maternal pre- or early pregnancy BMI and at least 1 offspring measurement (birth weight or childhood BMI)	Self-reported maternal and childhood BMI	Excessive GWG on the development of offspring obesity	Childhood overweight/obesity was associated with higher maternal pre-pregnancy BMI and gestational weight gain. This association was stronger at later ages.
Litvak et al. [53]	Systematic review of longitudinal, observational studies, n = 21	Healthy pregnant women and offspring body size	Assessing dietary patterns, macronutrients, foods, and beverages.	Offspring body size from 6 months to 18 years	Following a balanced diet, during pregnancy, together with a reduction of refined carbohydrate intake showed a positive effect on offspring adiposity at between 6 and 18 months after birth. Inconclusive or null findings associations of n-3 polyunsaturated fatty acids, protein, SSB artificially sweetened beverage intake and offspring body size were found.
Dodd et al. [54]	Randomized clinical trial, n = 2212	Women with a singleton pregnancy, between 10 + 0 and 20 + 0 weeks’ gestation, and BMI ≥25.	A comprehensive dietary and lifestyle intervention vs. standard care	Incidence of infants born large for gestational age (birth weight ≥90th centile for gestation and sex).	Overweight or obese women assigned to the intervention group did not reduce the risk of delivering large-for-gestational-age babies in comparison to the control arm (RR = 0.90; 95% CI 0.77–1.07; *p* = 0.24).
Phelan et al. [55]	Randomized clinical trial, n = 132	Healthy pregnant women at gestational age between 10 to 16 weeks	Intervention based on promoting a healthy weight gain by dietary and physical activity advice	Impact of excessive gestational weight gain, maternal eating and exercise on offspring weight status	High intake of sugar-rich foods was associated with large-for-gestational-age infants (β = 0.19, *p* = 0.004), macrosomia (OR = 1.1; 95% CI 1.0–1.2) and high birth weight (<90th percentile at birth) (OR = 1.2; 95% CI 1.1–1.3).
Chen et al. [56]	Cohort study, n = 910	Asian mother–child dyads	24h recall	Infant BMI	Higher maternal intake of SSBs was associated with higher offspring BMI z score at 24 and 48 months of age (0.07 SD; 95% CI 0.02–0.12 and 0.05 SD; 95% CI 0.004–0.09 respectively).
Quah et al. [57]	Cohort study, n = 1247	Asian mother–child dyads	Self-administered FFQ	Adiposity measures (BMI and skinfold thickness) and overweight/obesity status in children at 6 years of age.	An increment of 100 mL/day of SSB intake was associated with higher BMI (0.09 SD units; 95% CI 0.02–0.16), higher sum of skinfold thickness (0.68 mm; 95% CI 0.06–1.44) and increased risk of overweight/obesity (OR 1.2; 95% CI 1.07–1.23) at age 6 years.
Gillman et al. [58]	Prospective cohort study, n = 1078	Massachusetts mother–child dyads	FFQ	Childhood BMI, FMI and waist circumference	Maternal SBB intake during pregnancy was associated with higher BMI z scores (0.07 U; 95% CI −0.01–0.15), FMI (0.15 kg/m^2^; 95% CI −0.01–0.30) and waist circumference (0.65 cm; 95% CI 0.01–1.28).
Jen et al. [59]	Prospective cohort study, n = 3312	Netherland mother–child dyads	FFQ	Children BMI trajectories and body composition parameters	Maternal SSB intake during pregnancy was associated with higher BMI at ≤6 years of age children (per SSBs serving per day: 0.04 SD score; 95% CI 0.00–0.07).
Hu et al. [60]	Prospective cohort study, n = 1257	Tennesseans healthy mother–child dyads	The Block FFQ	Offspring growth and overweight/obesity risk from birth to age four years	Maternal dietary patterns rich in fried foods and SSBs were associated with higher risk of increase the BMI during growth (OR = 1.32; 95% CI 1.07–1.62) and higher risk of becoming overweight/obese children at 4 years of age (OR = 1.31, 95% CI 1.11–1.54).
Azad et al. [61]	Cohort study, n = 3033	Canadian mother–infant dyads	Modified FFQ to address usual food intakes during pregnancy	Infant BMI in the first year of life	Daily consumption of ASBs was associated with a 0.20-unit increase in infant BMI z score (95% CI 0.02–0.38) and a 2-fold higher risk of overweight at 1 year of age (adjusted OR = 2.19; 95% CI 1.23–3.88).
Zhu et al. [62]	Cohort study, n = 918	Danish mother–singleton child dyads in pregnancies complicated by gestational diabetes mellitus	Self-administrated FFQ	Offspring growth and the risk of overweight/obesity in childhood.	ASB intake during pregnancy was positively associated with offspring large-for-gestational age and overweight/obesity at 7 years compared to never consumption (adjusted RR = 1.57; 95% CI 1.05–2.35 at birth and adjusted RR = 1.93; 95% CI 1.24–3.01 at 7 years)
Cohen et al. [63]	Cohort study, n = 1234	Pregnant women and children aged 3.3 to 7.7 years	Self-administered FFQ	Child with child cognition outcomes	Excessive sugar intake (mean 49.8 g/day) was associated with reduced cognitive skills in children, as in the mid-childhood Kaufman Brief Intelligence Test (KBIT-II), non-verbal scores and early or mid-childhood scores.
Bédard et al. [64]	Longitudinal study, n = 8964	Pregnant women and children aged 7 to 9 years	FFQ	Respiratory and atopic outcomes	Higher mater intake of simple sugar during pregnancy increased the risk of atopic asthma (OR for highest vs. lowest quintile of simple sugar intake; OR = 2.01; 95% CI 1.23–3.29) and atopy (OR = 1.38; 95% CI 1.06–1.78).

AHEI: Alternate Healthy Eating Index diet; BMI: body mass index; ASBs: artificially sweetened beverages; CI: confidence interval; DASH: Dietary Approaches to Stop Hypertension diet; D+PA: hypocaloric Mediterranean type of diet and physical activity intervention; FFQ: food frequency questionnaire; FMI: fat mass index; GDM: gestational diabetes mellitus; Gestational hypertension: GHD; GWG: gestational weight gain; HR: hazard ratio; i-WIP: The International Weight Management in Pregnancy Collaborative Group; MedDiet: Mediterranean Diet; NHANES: National Health and Nutrition Examination; OR: Odds ratio; PA: physical activity intervention alone; PE: preeclampsia; PTD: preterm delivery; RCT: randomized control trial; SSB: sugar-sweetened beverage; RR: relative risk; TLC: Therapeutic Lifestyle Changes.
